# Infection of the Facial and Cervical Lymphatic Ganglia as a Result of Dental Lesions

**Published:** 1899-04

**Authors:** Chas. B. Porter


ARTICLE VIII.

### INFECTION OF THE FACIAL AND CERVICAL LYMPHATIC GANGLIA AS A RESULT OF DENTAL LESIONS.

CHAS. B. PORTER, D. D. S.

Read before the San Francisco Dental Association, Dec., 1898.

This subject was suggested by the case submitted to you at the last meeting, being a condition new to me; and since, from my limited experience, I cannot hope to find a subject of interest to the older members of the society, I must ask their tolerance and address myself to those who, like me, are but beginning.

In the case referred to the patient came to me in August and stated that a month earlier a tooth had abscessed and designated the right superior 6th year molar.

There was a large gold filling in the anterior ocluso-approximal portion faulty at tbe cervical border.

The tooth was of normal color but failed to give response to ice and to the hot blast from the chip blower.

The filling was removed and the pulp chamber entered with a bur and found to be absolutely empty and dry as were the canals in the palatal and disto-buccal roots.

The mesio-buccal root was impenetrable beyond one-eighth inch and was opened with fifty per cent. solution of sulfuric acid and a small Donaldson cleanser.

No pus was met but there was a strong odor of hydrogen sulfid. The usual treatments were made as indicated and the canals apparently rendered aseptic and filled with chloropercha and the cavity filled with gutta-percha.

Two weeks later the patient returned with a small "lump" in the substance of the cheek which was recognized as an inflamed lymphatic ganglion.

The canals were at once opened by use of oil of eucalyptus and a cleanser and treatment resumed.

The ganglion was at this time the size of a small bean and quite painful under pressure. Counter-irritation had no effect and the patient being a homœopath, was referred to her physician for constitutional treatment, without result.

Artificial communication was obtained with the apices of the buccal roots and medicines forced through.

In the meantime the buccal ganglion had enlarged to the size and shape of a large almond and the submaxillary ganglia had become involved and increased to the size of a hen's egg, and were very painful to pressure. A sinus developed opposite the disto-buccal root, had closed and the gum became much swollen.

No pus was found upon incision. Judging the abscessed roots to be the cause and finding the conditions did not yield, the tooth was extracted and the roots found much absorbed.

The socket was dressed and packed daily for several days when the wound seemed to heal comfortably.

To secure resolution of the ganglia a twenty per cent. aqueous solution of Ichthyol was applied to the inside of the cheek upon a lambswool tampon several times daily but with little or no effect.

About a week since, the gum appeared slightly inflamed and two sinuses developed.

Under an injection of three per cent. solution of Eucain the gum was dissected back and a carious area exposed embracing the entire socket.

The carious bone was removed with a large rose bur and the wound packed with Iodoform gauze and has since been washed out with Pyrozone followed by listerine and packed daily and seems to be progressing well.

Meanwhile an ointment of Ichthyol and Lanolin has been applied externally to the swellings, being rubbed in, while more spread upon lintine is bound on and allowed to remain over night with apparently good effect.

Not having met the condition before, I have been interested in looking up reports of cases. Mr. Bodecker says in his Anatomy and Pathology of the Teeth : "Inflammation of the Lymph ganglia is most common in submaxillary region, always due to the transmission of infectious material through lymphatic vessels into the ganglia. There are persons who, upon the slightest nonpurulent inflammation of the pulp or pericementum react with a swelling of the submaxillary ganglia.

Purulent pulpitis or pericementitis with all the inflammatory complications dependent upon them, are invariably accompanied by a swelling and hardening of the lymphatic ganglia."

In spite of this apparent frequency of occurrence, I can find but few cases reported, in none of which the ganglia required other treatment than the extraction of the tooth or cure of abscess causing the trouble.

As to the area involved, Mr. Bodecker says referring to the above-quoted paragraph : "All this holds good only for infectious diseases of the lower jaw, while those of the upper jaw rarely effect the submaxillary ganglia."

He makes no reference to affection of buccal, parotid or occipital ganglia. He says the condition is more common in youths under 10 years than in persons of more advanced age in whom it most frequently occurs during difficult eruptions of 3d molars or after their extraction and that mild cases sometimes occur after an attempted extraction of a tooth or root.

Bearing upon the relation between the condition of the oral cavity and these ganglia and the importance of the early recognition and prompt removal of the cause and always of prophylaxis, I shall read an extract from a paper by Dr. W. H. Bergtold of Buffalo. The paper is entitled, "The Mouth As a Center of Infectis." and of the Lymphatics he says—(Vol. XXXIV, page 124). We must therefore recognize the fact that the reflex inflammatory processes in these ganglia rarely terminate in suppuration, there is always its possibility, particularly in children, all of whom, according to Prof. Holt of the New York Polyclinic have a tendency toward lymphatism; in some so marked as to deserve to be classed as a distinct diathesis, and active measures should be taken to check the inflammation and promote resolution whenever it manifests itself.—*Pacific Medico-Dental Gazette.*

